# Improving Musculoskeletal Care Quality With a Web-Based Clinical Decision Support System in UK Primary Care: Mixed Methods Cluster-Randomized Controlled Trial

**DOI:** 10.2196/89734

**Published:** 2026-10-15

**Authors:** Jonathan Hill, Hollie Birkinshaw, Muhammed Usman, Tammy Millward, James Bailey, Simon Wathall, Rebecca Naylor, Robert Malcolm, Melissa Pegg, Hayden Holmes, Kate Lee, Tanya Wright, Adrian Chudyk, Alex Braybrooke, Roanna Burgess, Martyn Lewis

**Affiliations:** 1 School of Medicine, Keele University, David Weatherall Building, Stoke on Trent, Staffordshire, ST55BG, United Kingdom, 44 01782 733900; 2 York Health Economics Consortium, York, United Kingdom

**Keywords:** general practice, clinical decision support systems, musculoskeletal, cluster trial, randomized controlled trial, mixed methods, primary care, health technology.

## Abstract

**Background:**

Musculoskeletal (MSK) conditions account for one in seven general practice consultations in the United Kingdom. Despite national guidelines, suboptimal care remains common. Digital clinical decision support systems (CDSSs) offer a promising route to improve care quality but have not been robustly evaluated in MSK primary care.

**Objective:**

This study aimed to evaluate the efficacy, clinical effectiveness and cost-effectiveness, carbon reduction, safety, and implementation of a CDSS (CrossCover) for MSK consultations in UK general practice.

**Methods:**

We conducted a mixed methods, cluster-randomized controlled trial in 19 general practices between February and July 2024. Practices were randomized 1:1, stratified by list size. Intervention practices received training and access to the web-based CDSS for 3 months. We analyzed anonymized electronic health records to identify all adult MSK consultations. The primary outcome was the proportion of patient records containing one or more of 12 predefined non–evidence-based management options, assessed by blinded clinical review of 120 patient records (60 per arm). Reviewers assessed the complete 3-month record in both arms; in the intervention arm, suboptimal management was counted only where it occurred at a consultation in which the CDSS was used. Secondary outcomes included intention-to-treat treatment comparisons of self-management advice, prescriptions, referrals, and imaging. Clinician engagement, safety, environmental and economic modeling, and clinician experiences from semistructured interviews were evaluated. Outcome assessors were blinded; clinicians were not.

**Results:**

Among 10,182 eligible patients with MSK conditions (4268 intervention; 5914 control), the CDSS was used in 365 (8.6%) patient cases. Intention-to-treat analysis identified that the intervention increased self-management alone. Per-protocol analysis demonstrated that when used, suboptimal care arose in 3% (2/60) of CDSS-guided consultations vs 25% (15/60) of control consultations (adjusted odds ratio 12.30, 95% CI 2.52‐59.9; *P*=.002). It also increased self-management advice and community referrals, and it reduced prescribing and fit-note use. Safety concerns were logged in 6.6% (24/365) of uses with no high-risk issues identified. Environmental modeling estimated an 18% reduction in carbon emissions and 10% annual cost savings if the intervention were scaled. Interviews (n=18) highlighted positive perceptions of the CDSS’s content and safety netting but noted challenges in integration.

**Conclusions:**

When used, the CrossCover CDSS significantly reduced suboptimal primary care MSK management. However, while safety and clinician feedback were favorable, overall engagement was limited, underscoring the need for improved digital integration, workflow alignment, and implementation support.

## Introduction

Musculoskeletal (MSK) conditions represent a significant burden for health systems, accounting for approximately one in seven general practice consultations in the United Kingdom [1,2]. Onward referral decisions are often complex and, if misdirected, can lead to avoidable imaging, unnecessary outpatient appointments, and patient dissatisfaction. Despite national guidelines, there remain large and persistent gaps in the quality of MSK care. Primary care data indicate underuse of nonpharmacological treatments and high rates of opioid and gabapentinoid prescribing, often locking patients into cycles of repeat attendance and low-value care [3-5]. Other examples of suboptimal management include long-term use of nonsteroidal anti-inflammatory drugs without gastroprotection, repeat steroid injections at short intervals, unwarranted imaging, and frequent issuance of fit notes without vocational support [6-8].

Improving the consistency and quality of early MSK care is a national priority, with implications for patient outcomes, health care sustainability, and elective recovery efforts [9]. One proposed solution is the use of digital clinical decision support systems (CDSSs), which aim to embed evidence-based guidance into routine consultations in real time [10]. While systematic reviews show CDSSs can improve diagnostic accuracy, referral quality, and guideline adherence across various clinical domains [11,12], their effectiveness in MSK care specifically remains unclear. Successful adoption of CDSSs has also been shown to depend on co-design with clinicians, seamless workflow integration, and appropriate training and support [13].

Within MSK primary care, very few CDSS evaluations have been conducted. The Norwegian SupportPRIM trial was underpowered and showed no effect on clinician behavior or patient outcomes [14], while the STarT MSK trial—which focused on risk-based stratification—also failed to demonstrate improvements in outcomes or care delivery [15-17]. Other alternatives to general practitioner (GP)-led MSK management include first contact practitioners (FCPs), direct access to triage clinics, and “Advice and Guidance” platforms. These models show promise [18-22], but are variably implemented and face concerns over workforce sustainability, clinical consistency, and GP workload.

CrossCover, a UK-based digital health company, developed the Orthopathway CDSS (version 4), designed to support MSK decision-making in general practice. At the time of trial launch (early 2024), the tool included 8 clinician-facing MSK pathways that were integrated with the EMIS and SystmOne electronic health record (EHR) systems. The CDSS was co-designed by a local multidisciplinary team, while CrossCover handled digital integration, referral automation, and system feedback for reporting safety concerns and improvement suggestions. Preliminary internal evaluation showed reduced unnecessary referrals and investigations, prompting the need for formal evaluation [23,24].

This study therefore aimed to evaluate whether embedding a CDSS into routine UK general practice consultations could reduce nonevidence-based MSK management. Using anonymized EHR data, we also examined impacts on treatment patterns (eg, prescriptions, referrals, and imaging) as indicators of both efficacy when used and effectiveness across all consultations, in addition to clinician engagement, safety reporting, and modeled environmental and economic outcomes. Qualitative interviews were conducted to explore implementation experiences among both clinicians and patients.

## Methods

### Study Design and Setting

We conducted a pragmatic, mixed methods, cluster-randomized controlled trial with an embedded qualitative study in 19 general practices across the West Midlands, United Kingdom (10 intervention, 9 control) in early 2024. A cluster design was necessary because, once installed, the CDSS became accessible to all clinicians in the practice. Individual-level randomization was therefore not feasible due to the risk of contamination [25]. The trial protocol is publicly available [26]. Practices were recruited between February and April 2023, and randomized in a single batch in January 2024. The intervention period ran from February to June 2024, with each practice contributing a 3-month recruitment window plus 3 months of follow-up EHR data. Qualitative interviews were conducted following the intervention period, between June and August. Data extraction and analysis were completed in May 2025.

Participating clinicians included GPs and FCPs. GP practice involvement, consent, licensing, and training were supported by the National Institute for Health Research Clinical Research Network and a local health care team from the Birmingham and Solihull Integrated Care Board.

The trial is reported in accordance with the CONSORT-EHEALTH (Consolidated Standards of Reporting Trials of Electronic and Mobile Health Applications and Online Telehealth) guidelines [27], and the completed CONSORT-EHEALTH checklist (version 1.6.1) is provided in Checklist 1.

### Protocol Deviations and Amendments

The original trial protocol included patient-reported outcome measures data collection via a trial-specific computer prompt during consultations. However, it failed to operate as intended, resulting in low recruitment rates. Following this, an ethics amendment was approved to abandon patient-reported outcome measure collection and instead rely solely on anonymized EHR data for all outcomes and to change the primary outcome to care quality.

### Randomization and Blinding

The unit of allocation and intervention within this cluster-randomized trial was the GP practice. GP practices were randomized 1:1 by an independent statistician using stratified randomization based on registered patient list size. Due to the nature of the intervention, clinician blinding of clinicians was not feasible. Patients were not informed of group allocation. The study used routinely collected, anonymized EHR data, and the Health Research Authority approved processing of these data without individual patient consent (IRAS 326002; REC 23/YH/0105). Patients who had opted out of data sharing for research were excluded. Outcome analysis was performed by a statistician blinded to group allocation.

### Sample Size

The trial was powered to detect a 20% absolute reduction in nonevidence-based management options in the intervention arm, assuming a baseline proportion of 40%. With an intracluster correlation coefficient (ICC) of 0.01 and a 2-tailed α of .05, a total of 400 patients (200 in each arm; 20 per cluster) provided more than 90% power for superiority testing. For the primary outcome, a random subsample of 120 patient records (60 per arm) was selected. Using PASS 2022 (NCSS, LLC) and the procedure for testing 2 proportions in a cluster-randomized design (2-sided unpooled *Z*-test), we estimated power for a design of 10 clusters per arm with 6 records per cluster (60 per arm). Assuming proportions of 0.625 and 0.375 in the 2 arms (a 25 percentage-point absolute difference), an ICC of 0.01, and a 2-sided α of .05, this provided approximately 79% power. The assumed proportions were set close to 0.5, where the binomial variance and hence the required sample size are greatest, giving a conservative estimate [25]. In the event, the sampled records came from 7 practices per arm with a mean of 8.6 records per practice. At an ICC of 0.01, the larger cluster size has a negligible effect on the design effect, but estimating between-practice variance from 7 rather than 10 clusters per arm reduces precision, so realized power was somewhat below the 79% estimated at the design stage. The observed proportion of control records containing suboptimal management (15/60, 25%) was lower than the 40% assumed for the trial-level calculation at the design stage, although the absolute between-arm difference observed (22 percentage points) was close to the 25 percentage points the subsample was powered to detect.

### Participant Eligibility and Recruitment

Inclusion criteria for the anonymized EHR analysis were consultations in adults aged ≥18 years with an eligible MSK code [28] during a 3-month trial window (February to July 2024, depending on practice onboarding). Patients who had opted out of data sharing for research were excluded. Clinicians were prompted to record pain intensity scores during MSK consultations. Qualitative participants included purposively sampled patients, clinicians from intervention practices, and secondary care specialist triage clinicians receiving CDSS-facilitated referrals.

### Interventions

The intervention was CrossCover Orthopathway CDSS [23], a UK Conformity Assessed (UKCA)-marked class 1 medical device featuring MSK pathways for the elbow, foot and ankle, hand and wrist, hip, knee, paediatrics, shoulder, and spinal pain. The pathways were co-designed by a local multidisciplinary team and built on national MSK guidance; clinical content for each body-region pathway was validated by consultant physiotherapists, orthopedic and rheumatology specialists, and GPs, while CrossCover delivered digital integration, referral automation, and the safety and feedback logging. A CDSS link appeared during a consultation after clinicians entered a relevant MSK diagnosis code into the EHR. Training was offered to all intervention clinicians, although typically a single clinician per practice cascaded training internally, as the CDSS was new and in the process of being adopted locally at the time of the trial. No changes were made to the orthopathway intervention during the study period. There were no software updates, bug fixes, content or functionality changes, or system downtime that affected the delivery of the intervention.

Control practices delivered usual care with no restrictions on the information sources clinicians might use; the only trial-specific addition was a prompt to record pain scores.

### Outcomes

#### Primary Outcome

The primary outcome was a per-protocol (PP) analysis that used anonymized EHR data from consultations where the CDSS was used over a 3-month period to assess the frequency of 12 prespecified nonevidence-based MSK management options. This outcome was selected because it directly reflects the CDSS’s intended effect: reducing low-value or nonevidence-based care.

The 12 suboptimal management options (Multimedia Appendix 1) were identified prior to data analysis and were specifically developed for the trial. They were derived from our published systematic review of primary-care and community-care MSK quality indicators [29] and refined for feasibility within routine UK EHR data. Each indicator maps to an evidence-based recommendation from current national or international guidance. For example, avoidance of imaging for nonspecific low back pain without red flags (NICE [National Institute for Health and Care Excellence] NG59) [30], gastroprotection with long-term nonsteroidal anti-inflammatory drugs (NICE NG226), and avoidance of opioids and gabapentinoids for persistent MSK and low back pain (NICE NG59, NG193, and NG226). The composite was prespecified, binary at the patient-record level (a record was flagged if one or more indicators were present at an assessable consultation), and all indicators were weighted equally a priori.

The blinded review judged the appropriateness of primary-care decisions (prescribing, imaging, referral, and safety-netting) against prespecified primary-care quality indicators. Therefore, the panel was deliberately composed of clinicians who make these decisions and have appropriate clinical contextual knowledge: an experienced GP (the decision-maker), a physiotherapist with MSK specialist expertise, and a clinical researcher for methodological consistency. No automated rules were applied.

To standardize interpretation of the indicators, the panel scored the first 10 records and then reviewed their scoring together, with particular attention to indicators involving clinical judgment, such as repeated attendance without adequate management. The remaining 110 records were scored independently. Interrater agreement was calculated for these 110 records, after the calibration exercise and before any adjudication, and was complete (Fleiss κ 1.0). No disagreements arose, so the prespecified third-reviewer adjudication process was not invoked. Complete agreement is uncommon in record review and should be interpreted in the context of the calibration exercise and of the fact that most indicators, such as imaging requests, prescription duration, and referral destination, are directly verifiable from the record.

The blinded reviewers assessed the complete 3-month record for every sampled patient in both arms so that indicators requiring more than one contact, such as repeated attendance or repeat fit notes, could be judged in context. In the control arm, suboptimal management was recorded wherever it occurred within that window. In the intervention arm, it was recorded only when it occurred at a consultation in which the CDSS was used, judged against all preceding contacts. This PP definition was adopted because patients were frequently seen more than once within the window by different clinicians, only some of whom used the CDSS, and management at a consultation where the tool was never opened cannot reasonably be attributed to it. Reviewers recorded the consultation at which each instance of suboptimal management occurred while blinded to allocation and to CDSS use, and CDSS status was linked to those consultations subsequently by the trial data manager. A sensitivity analysis assessing all consultations in both arms is reported alongside the primary analysis.

#### Sampling for the Primary Outcome

The random subsample was drawn in SQL by the trial data manager, who took no part in the blinded review. In the control arm, the sampling frame was all 5914 eligible patients across the 9 control practices. In the intervention arm, it was the 365 patients for whom the CDSS was used at least once, across the 8 practices in which it was used. Sixty patient records were selected at random from each frame, without stratification by practice. Because eligible patient numbers varied widely between practices, the smallest practices had a substantial probability of contributing no records to a subsample of this size.

#### Secondary Outcomes

Secondary outcomes included an intention-to-treat (ITT) analysis to compare analgesic prescriptions, urgent and routine specialist referrals, community referrals, imaging requests, blood tests, steroid injections, self-management and exercise advice, and the issuance of fit notes (UK certification for work absence due to illness).

A before-and-after sensitivity analysis used aggregated secondary care data to assess whether differences in orthopedic referral rates between trial arms reflected historical patterns rather than the impact of the CDSS (the specialty was chosen for its high volume/cost). Crude referral rates (not adjusted for age or sex) were calculated for 2023 (preimplementation) and 2024 (postimplementation) and compared by GP practice and randomized group.

### Clinician Engagement

Clinician engagement with the CDSS was assessed using in-platform analytics. Metrics included the proportion of eligible patients for whom the tool was used, the average time spent using it, the number and proportion of uses by GPs and FCPs, and the rate at which users opted out of the CDSS’s treatment recommendations.

### Safety Assessment

Clinicians could report safety concerns using an in-platform issue log. Reported issues were reviewed and categorized independently by members of the academic trial team, without input from the CDSS industry partner. Using a structured classification framework [31], each issue was grouped by its likely cause (such as data entry errors, algorithm inconsistencies, user interface design, or system integration problems), type of impact (eg, accuracy or timeliness), and severity of risk of patient harm (low, moderate, or high).

### Environmental and Economic Impact Substudy

A validated carbon footprint and cost calculator was used to estimate resource use across 63 common MSK management options [5]. Health care utilization data were extracted using SNOMED CT-coded EHRs data and standardized methods [28], across primary, community, and secondary care settings [5]. While the full methodology and results of this substudy are reported elsewhere, key overall findings are included here to support broader impact interpretation. Costs are reported in pounds sterling (£) with conversions to US dollars at the 2024 average exchange rate of £1 = US $1.28.

### Data Analysis

All quantitative analyses were performed using Stata v17 (StataCorp LLC). The PP primary analysis used hierarchical (multilevel) generalized linear regression models to examine treatment differences across groups, adjusting for practice list size (fixed effect) and clustering by GP practice (random effect). Binary outcomes used logit links, while count outcomes used negative binomial models to account for overdispersion. The primary analysis tested for superiority using a 2-sided 5% α, with 95% CIs.

Two secondary comparisons were performed in relation to the scenarios of CDSS use within the trial. First, the ITT analysis compared participants in intervention vs control practices to explore the effectiveness of the CDSS across all consultations, whether the CDSS was used or not. Second, a PP analysis compared consultations in which the CDSS was used with control consultations to explore the efficacy of the CDSS when used. A post hoc sensitivity analysis used cluster-level modeling to test the robustness of the findings, given the relatively small number of clusters [32].

### Qualitative Substudy

A qualitative study was embedded within the trial to explore clinician experiences of the CDSS. The full methodology and results are reported in a separate manuscript (Lee K, Wright T, Birkinshaw H, Hill JC, unpublished data, 2026). However, to support interpretation of clinician intervention engagement, selected findings are summarized here. Semistructured interviews were conducted online via Microsoft Teams and audio-recorded. A topic guide was developed by the study team and refined during data collection. Transcripts were anonymized and analyzed using reflexive thematic analysis [33], with coding supported by NVivo software (Lumivero). We reported the qualitative methods and findings in accordance with SRQR (Standards for Reporting Qualitative Research) guidelines [34].

### Ethical Considerations

Ethical approval was granted by the Health Research Authority on June 8, 2023 (IRAS project ID: 326002; REC reference: 23/YH/0105). Written, informed consent was obtained from all qualitative participants.

## Results

### Study Population

Of the 30 GP practices approached, 20 provided final consent and were randomized; 19 actively participated in the trial (10 intervention, 9 control) (Figure 1). Across these 19 practices, 10,182 unique adult patients consulted for an MSK pain condition during the study period (4268 in intervention practices, 5914 in control practices).

**Figure 1.**
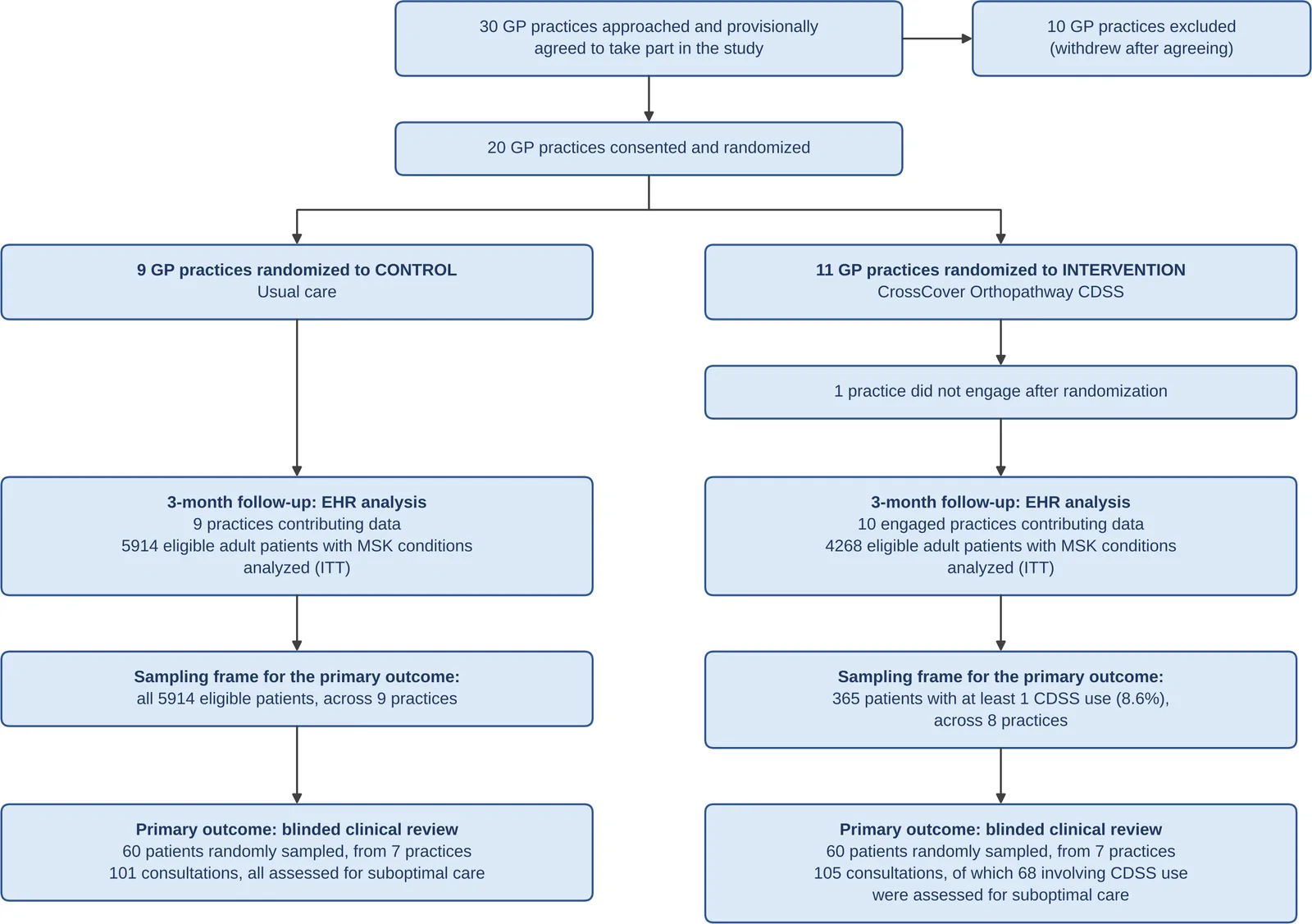
Flow of general practices and patients through the trial, from practice recruitment to derivation of the primary outcome sample in each arm. CDSS: clinical decision support system; EHR: electronic health record; GP: general practitioner; ITT: intention-to-treat; MSK: musculoskeletal.

The CDSS was used in 365 (8.6%) intervention patients, involving 56 clinicians across 8 of the 10 intervention patient practices, and there were 397 uses across those 365 patients. Patients had a mean age of more than 50 years; 59.0% (6005/10,182) were female. The most common presenting complaints were spinal, knee/leg, and multisite pain. Baseline characteristics are summarized in Table 1.

**Table 1.** Baseline characteristics split by treatment group.

Characteristic	Control (practices: n=9; patients: n=5914)	Intervention (practices: n=10; patients: n=4268)	Subgroup of patients in whom CDSS^a^ was used at least once (practices: n=8; patients: n=365)
Age (y), n; mean (SD)	5902; 51.9 (19.5)	4245; 52.7 (19.8)	365; 54.6 (19.2)
Pain intensity, n; mean (SD)	408; 6.3 (2.2)	261; 6.7 (2.1)	31; 6.9 (1.9)
Sex, n (%)			
Female	3529 (59.7)	2476 (58.0)	211 (57.8)
Pain location, n (%)			
Ankle/foot	657 (11.1)	411 (9.6)	33 (9.0)
Arm/elbow	213 (3.6)	145 (3.4)	12 (3.3)
Back/spine	1344 (22.7)	836 (19.6)	80 (21.9)
Hand/wrist	520 (8.8)	382 (9.0)	32 (8.8)
Hip	324 (5.5)	222 (5.2)	30 (8.2)
Leg/knee	1081 (18.3)	705 (16.5)	82 (22.5)
Multisite	1007 (17.0)	954 (22.4)	23 (6.3)
Neck	243 (4.1)	181 (4.2)	26 (7.1)
Shoulder	445 (7.5)	342 (8.0)	47 (12.9)
Not specified	80 (1.4)	90 (2.1)	0 (0.0)

a CDSS: clinical decision support systems.

### Primary Outcome

Table 2 presents the results of the blinded clinical record review; interrater agreement on the 110 independently reviewed records was complete (Fleiss κ 1.0). The sampled records were drawn from 7 practices in each arm. The 60 control records comprised 101 consultations, all of which were assessed. The 60 intervention records comprised 105 consultations, of which 68 (64.8%) involved CDSS use and were therefore assessable under the PP definition.

**Table 2.** Appropriateness of musculoskeletal care in the randomly sampled patient records and associated consultations.

Measure	Control	Intervention	OR^a^ (95% CI)^b^	*P* value
Sample, n				
Practices represented^c^	7	7	—^d^	—
Patients randomly sampled	60	60	—	—
Consultations in the 3-month window	101	105	—	—
Consultations assessed for suboptimal care^e^	101	68	—	—
Consultations not assessed (CDSS^f^ not used)	0	37	—	—
Primary analysis: suboptimal care attributed to consultations where the CDSS was used				
Received appropriate care, n (%)	45 (75.0)	58 (96.7)	—	—
Patients with suboptimal care, n (%)	15 (25.0)	2 (3.3)	—	—
Suboptimal care per assessed consultation, n/N (%)	15/101 (14.9)	2/68 (2.9)	—	—
Adjusted for practice size, age, and sex	—	—	12.30 (2.52‐59.90)	.002
Sensitivity analysis: all consultations assessed in both arms				
Received appropriate care, n (%)	45 (75.0)	52 (86.7)	—	—
Patients with suboptimal care, n (%)	15 (25.0)	8 (13.3)	—	—
Suboptimal care per assessed consultation, n/N (%)	15/101 (14.9)	8/105 (7.6)	—	—
Unadjusted	—	—	2.16 (0.84‐5.57)	.11
Adjusted for practice size	—	—	2.54 (0.96‐6.68)	.06
Adjusted for practice size, age, and sex	—	—	2.74 (1.00‐7.48)	.05

a OR: odds ratio.

b Odds ratios are for receiving appropriate care in the intervention group relative to the control group, estimated by multilevel logistic regression with practice as a random effect. Values above 1 favor the intervention.

c Practices were represented among the 60 randomly sampled patients in each arm. The sampling frames comprised 9 control practices and the 8 intervention practices in which the CDSS was used.

d Not applicable.

e Blinded reviewers assessed the complete 3-month record for every sampled patient in both arms. In the intervention arm, suboptimal care was attributed only when it occurred during a consultation in which the CDSS was used; see *Methods *section.

f CDSS: clinical decision support system.

Suboptimal management was identified in 2 of 60 (3%) intervention records and 15 of 60 (25%) control records. Adjusted analysis indicated a significantly lower likelihood of suboptimal care in the intervention group (adjusted odds ratio (OR) 12.30, 95% CI 2.52‐59.90; *P*=.002). When expressed per assessed consultation, suboptimal management was identified in 2 of 68 (3%) CDSS-guided consultations, compared with 15 of 101 (14.9%) control consultations.

In a sensitivity analysis assessing all consultations in both arms (ie, intervention consultations not restricted to CDSS use), suboptimal management was identified in 8 of 60 (13%) intervention records, giving an adjusted OR of 2.74 (95% CI 1.00‐7.48; *P*=.05). The direction of effect was consistent across both definitions, although the difference was borderline significant when management at intervention consultations where the CDSS had not been used was included.

Among the 37 intervention consultations in which the CDSS was not used, suboptimal management was identified in 6 (16%). This was closely comparable to the rate in control consultations (15/101, 14.9%) and several times higher than in consultations where the CDSS was used (2/68, 3%). Care delivered within intervention practices when the tool was not opened was therefore indistinguishable from usual care, which suggests the difference observed is consistent with an effect of CDSS use rather than underlying differences between practices. This comparison was conducted post hoc at the request of peer reviewers.

Findings in the control arm were concentrated in 2 indicators: multiple attendances with only a prescription issued and no documentation of advice, investigation or referral (5 vs 0), and spinal imaging for low back pain without red flag indicators (4 vs 0). Together these accounted for 9 of the 15 instances identified in control records. Across the prescribing and injection indicators, counts were similar or slightly higher in the intervention arm. A full breakdown by indicator under both definitions is provided in Multimedia Appendix 1.

### Secondary Outcomes

Table 3 presents secondary outcomes from the ITT analysis that explored effectiveness in all consultations. No statistically significant differences were found between groups, except for a higher rate of recorded self-management and exercise advice in the intervention group. A before-and-after sensitivity analysis of orthopedic referral rates, using secondary care data, showed no material differences between groups in either the preimplementation or postimplementation period, supporting the robustness of the main findings.

**Table 3.** Comparison of general practitioner (GP) decision-making per group (0‐90 d) using anonymized electronic health record (EHR) data: ITT analysis.

Management decision	Control (practices: n=9; patients: n=5914)		Intervention (practices: n=10; patients: n=4268)		ITT^a^ comparison (main analysis)^b^		ITT comparison (sensitivity)^c^	
	Patients, n (%)^d^	Events, n (rate per patient)^e^	n (%)	Count (mean)	OR^f^ (95% CI)^g^	RR^h^ (95% CI)^g^	MD^i^ (95% CI)^j^	MD (95% CI)^k^
Analgesic prescription^l^	1887 (31.9)	12,468 (2.11)	1458 (34.2)	7677 (1.80)	—^m^	0.72 (0.42-1.23)	−6.5 (−23.8 to 10.7)	−4.6 (−21.1 to 12.0)
Urgent specialist referral	214 (3.6)	240 (0.04)	88 (2.1)	104 (0.02)	0.84 (0.44-1.59)	—	−0.3 (−1.9 to 1.3)	−0.8 (−2.2 to 0.6)
Routine specialist referral	380 (6.4)	430 (0.07)	183 (4.3)	191 (0.05)	1.85 (0.22-15.5)	—	0.5 (−5.8 to 6.8)	−4.2 (−9.2 to 0.8)
Community referral	214 (3.6)	258 (0.04)	224 (5.3)	230 (0.05)	2.49 (0.34-18.0)	—	2.4 (−3.6 to 8.3)	−0.3 (−5.0 to 4.3)
Imaging request^n^	244 (4.1)	266 (0.05)	278 (6.5)	319 (0.08)	1.60 (0.72-3.52)	—	1.5 (−2.3 to 5.3)	1.5 (−2.1 to 5.2)
Blood test	903 (15.3)	1390 (0.24)	931 (21.8)	1394 (0.33)	—	0.74 (0.16-3.54)	−9.3 (−34.3 to 15.7)	−4.0 (−24.0 to 16.0)
Steroid injection	405 (6.9)	508 (0.09)	296 (6.9)	365 (0.09)	1.11 (0.36-3.41)	—	0.3 (−5.4 to 6.0)	1.1 (−3.7 to 5.9)
Information/exercise/advice	1005 (17.0)	1262 (0.21)	1218 (28.5)	1527 (0.36)	—	2.19 (1.01-4.75)^o^	13.6 (−0.5 to 27.7)	14.5 (2.2 to 26.9)^m^
Fit-note certification	676 (11.4)	1239 (0.21)	370 (8.7)	670 (0.16)	—	0.84 (0.32-2.18)	−0.6 (−7.8 to 6.7)	−1.8 (−8.3 to 4.7)

a ITT: intention-to-treat.

b Main analysis (generalized linear mixed modeling): statistical testing was carried out using a multilevel logistic regression model for binary outcome data (giving OR estimation) and a multilevel negative binomial regression model for count data (with RR estimation), including practice as a random effect and adjusting for practice size as a fixed effect.

c Sensitivity analysis (cluster-level analysis [CLA]): statistical testing was carried out using multiple linear regression for the mean difference (MD) in cluster-level proportions of patients, with adjustment for the practice-size fixed effect.

d Number and percentage of patients with at least one management decision recorded in the 0‐90 day window.

e Total number of events recorded across all patients in the arm, with the rate per patient (events divided by patients in the arm); for example, 12,468 analgesic prescriptions among 5914 control patients gives 2.11 per patient. Count outcomes were compared using negative binomial regression (rate ratios) because the data were overdispersed.

f OR: odds ratio.

g OR and RR (relative scales) are for the intervention relative to the control.

h RR: rate ratio.

i MD: mean difference in proportions.

j Analysis is unweighted (equal weighting was given to all practices within the regression model).

k Analysis was weighted (inverse-variance weighting was used to weight practices according to spread and sample size within the regression model).

l Prescription of nonsteroidal anti-inflammatory drugs for more than 1 month without concurrent gastroprotection.

m Not applicable.

n Repeated imaging requests for nonspecific LBP without a clear clinical indication.

o Statistically significant at the 5% level (2-tailed); the 95% CI excludes 1 for OR and RR, or 0 for MD.

Table 4 presents the results from the PP analysis to explore the efficacy of the CDSS, comparing consultations where the CDSS was used (n=365) with controls. CDSS-guided care was associated with fewer prescriptions and fit notes and with more frequent urgent specialist referrals (a proxy for better red flag identification), community referrals, and provision of self-management advice. Post hoc sensitivity analyses at the cluster level supported these findings.

**Table 4.** Comparison of general practitioners’ (GP) decision-making per group (0‐90 d) using anonymized electronic health record (EHR) data: per-protocol (PP) analysis^a^.

Management decision	Control (practices: n=9; patients: n=5914)		Intervention (practices: n=8; patients: n=365)		PP comparison (sensitivity)^b^		PP comparison (main analysis)^c^	
	patients, n (%)^d^	Events, n (rate per patient)^e^	n (%)	Count (mean)	OR^f^	RR^g^	MD^h,i^	MD^h,j^
Analgesic prescription	1887 (31.9)	12,468 (2.11)	34 (9.3)	132 (0.36)	—^k^	0.13 (0.06-0.28)^l^	−29.2 (−46.9 to −11.5)^m^	−26.9 (−46.7 to −7.2)^m^
Urgent specialist referral	214 (3.6)	240 (0.04)	25 (6.9)	27 (0.07)	3.14 (1.57-6.30)^l^	—	5.3 (1.6 to 9.1)^m^	4.8 (2.2 to 7.5)^m^
Routine specialist referral	380 (6.4)	430 (0.07)	13 (3.6)	13 (0.04)	3.55 (0.55-22.8)^n^	—	2.0 (−3.7 to 7.6)	−2.2 (−7.4 to 3.0)
Community referral	214 (3.6)	258 (0.04)	75 (20.6)	77 (0.21)	20.0 (3.61-111)^l^	—	19.0 (6.2 to 31.8)^m^	15.5 (6.1 to 24.9)^m^
Imaging request	244 (4.1)	266 (0.05)	21 (5.8)	20 (0.05)	1.55 (0.55-4.36)	—	1.7 (−3.3 to 6.7)	2.2 (−3.2 to 7.7)
Blood test	903 (15.3)	1390 (0.24)	21 (5.8)	46 (0.13)	—	0.21 (0.03-1.51)	−22.7 (−45.6 to 0.2)	−7.1 (−39.3 to 25.2)
Steroid injection	405 (6.9)	508 (0.09)	28 (7.7)	34 (0.09)	1.37 (0.45-4.14)	—	0.5 (−5.6 to 6.5)	2.5 (−4.6 to 9.6)
Information/exercise/advice	1005 (17.0)	1262 (0.21)	325 (89.0)	351 (0.96)	—	7.07 (3.69-13.6)^l^	73.1 (62.8 to 83.5)^m^	77.2 (65.6 to 88.9)^m^
Fit-note certification	676 (11.4)	1239 (0.21)	4 (1.1)	7 (0.019)	—	0.08 (0.02-0.43)^l^	−8.2 (−14.8 to −1.6)^m^	−5.6 (−15.1 to 3.9)

a MSK consultations include face-to-face, telephone, or video consultations. OR and RR (relative scales) are for intervention relative to the control. MD (absolute scales) is for intervention minus control.

b Sensitivity analysis (cluster-level analysis [CLA]): statistical testing was carried out using multiple linear regression for the MD in cluster-level proportions of patients, adjusting for the fixed effect of practice size.

c Main analysis (generalized linear mixed modeling): statistical testing was carried out using a multilevel logistic regression model for binary outcome data (giving OR estimates) and a multilevel negative binomial regression model for count data (with RR estimation), including practice as a random effect and adjusting for practice size as a fixed effect.

d Number and percentage of patients with at least 1 management decision recorded in the 0‐90 day window.

e Total number of events recorded across all patients in the arm, with the rate per patient (events divided by patients in the arm); for example, 12,468 analgesic prescriptions among 5914 control patients gives 2.11 per patient. Count outcomes were compared using negative binomial regression (rate ratios) because the data were overdispersed.

f OR: odds ratio.

g RR: rate ratio.

h MD: mean difference in proportions.

i Analysis was unweighted (equal weighting was given to all practices within the regression model).

j Analysis was weighted (inverse-variance weighting was used to weight practices according to spread and sample size within the regression model).

k Not applicable.

l Statistically significant *P* values (*P*<.05).

m Indicate statistically significant between-group comparisons at the 5% 2-tailed significance level.

n The adjusted OR for routine specialist referral differs in direction from the crude proportions. With few clusters and low event prevalence, the multilevel estimate for this outcome is unstable, as reflected in the very wide CI, and should not be interpreted.

### Clinician Engagement

Clinician engagement with the CDSS was limited. Over the 3-month intervention period, there were 397 uses of the CDSS among 8.6% (365/4268) eligible patients. Use was uncommon in follow-up appointments. A total of 56 clinicians used the CDSS: 41 GPs and 15 FCPs. GPs accounted for 56% (221/397) of uses and FCPs for 44% (176/397). The mean use time per consultation was 4 minutes 39 seconds (SD 4.36), and a 5% opt-out rate was observed for CDSS-generated treatment recommendations.

### Safety

There were 24 safety log submissions in 365 (6.6%) uses of the CDSS, categorized as follows: no high-risk issues, 3 moderate-risk (1 algorithm labeling error and 2 integration issues), and 21 low-risk or system improvement suggestions. Overall, the CDSS was judged to be safe, with no reports of patient harm, and was seen as responsive to clinician feedback.

### Qualitative Findings

Semistructured interviews with clinicians (n=18) identified key barriers to CDSS use. GPs noted that its MSK-specific focus conflicted with the multimorbidity and time constraints typical of general practice consultations. The binary nature of outputs (eg, referral/no referral) was considered limiting for conditions requiring longer-term monitoring. Other barriers included the perceived learning curve for new software, concerns over growing reliance on algorithms, and the sense that the initiative prioritized secondary care goals over primary care needs. Participants emphasized the importance of improved integration, communication, and incentives to support uptake. Further qualitative results are reported in a separate manuscript.

### Environmental and Economic Impacts

Modeling suggested that the intervention could reduce annual carbon dioxide equivalent (CO₂e) emissions across Birmingham and Solihull Integrated Care Board by 940 tonnes (equivalent to 3.36 million petrol car miles) and generate annual cost savings of £1.6 million (US $2.05 million). The largest carbon contributor was prescribing, while the main cost driver was GP appointments. On a per-person basis, CDSS-guided care reduced mean emissions by 4.14 kg CO₂e and costs by £7.10 (US $9.09), representing an 18% reduction in emissions and a 10% reduction in costs. Full methodology and detailed results are reported in a separate publication (Naylor R, Malcolm R, Pegg M, et al, unpublished data, 2026).

## Discussion

### Principal Results

This mixed methods, cluster-randomized trial evaluated the CrossCover Orthopathway CDSS in over 10,000 MSK consultations in UK general practice. In a PP analysis, the intervention was associated with lower guideline nonadherent care: suboptimal management was recorded in 3% (2/60) of CDSS-guided consultations compared with 15/60 (25%) of control records. This estimate is imprecise: it rests on 2 events in the intervention arm and 15 in the control arm, and the CI around the OR is correspondingly wide. The difference was concentrated in 2 of the 12 indicators: repeated attendance without documented advice, investigation, or referral, and imaging for nonspecific low back pain without red flags. Across the prescribing and injection indicators, counts were similar or slightly higher in the intervention arm, which is consistent with the CDSS’s focus on referral and pathway decisions rather than on what to prescribe.

A post hoc comparison within the intervention arm provides some reassurance that this reflects the tool rather than differences between practices. Among intervention consultations in which the CDSS was not used, suboptimal management was identified at a rate closely comparable to that in control consultations and several times higher than in consultations in which the tool was used. Care delivered within intervention practices when the CDSS was not opened, therefore, resembled usual care.

In the ITT secondary outcome analysis, which reflects the real-world effectiveness of the CDSS, the intervention group also received more documented self-management and exercise advice. In the PP secondary outcome analysis, additional benefits included reduced analgesic prescribing and fit-note certification, alongside increased referrals to physiotherapy and urgent secondary care, consistent with the CDSS’s escalation logic for red-flag presentations, such as suspected cauda equina syndrome or fracture.

### Clinician Engagement

However, CDSS usage occurred in only 8.6% of eligible patients, reflecting persistent barriers to clinician engagement. This corroborates findings from other trials—including STarT MSK [35], where tools were perceived as disruptive to consultation flow. Low uptake is not unique to this intervention: a systematic review of CDSS uptake found that use is seldom reported and, where reported, is often low and identified this as a major and potentially modifiable barrier to overall CDSS effectiveness [36]. While user feedback in this study was largely positive, with an average usage time under 5 minutes and low opt-out rates, broader adoption remains a key challenge. Notably, usage appeared influenced by clinical role: GPs, whose caseloads span many conditions, encountered the CDSS less frequently, limiting habituation. In contrast, FCPs, whose caseloads are MSK-specific, engaged more frequently with the tool. Direct evidence linking caseload concentration to sustained CDSS engagement is limited, but this suggests that FCPs may represent a priority group for CDSS implementation in primary care.

### Comparison with Prior Work

Interventions aiming to change clinician behavior in MSK care have a mixed record. Single-component implementation efforts have consistently failed to shift low back pain practice, with multifaceted approaches appearing necessary [37], and behavioral nudging trials such as those led by Maher et al [17] have shown limited success. Clinical decision support systems (CDSSs) perform better, but modestly and selectively: across 122 trials involving over 1.2 million patients, CDSSs increased the proportion of patients receiving the specific care targeted by a tool by 5.8% (95% CI 4.0%‐7.6%), with marked heterogeneity between targets and a median improvement of only 0.3% in clinical end points [38]. Effects concentrate on the decisions a tool actually prompts rather than generalizing across care [39].

That pattern helps explain the shape of our findings. Orthopathway addressed referral and pathway decisions, and it was in referral and imaging that differences emerged; it offered little guidance on analgesic choice or the timing of joint injections, and in those domains we found no benefit. Guideline-discordant imaging for low back pain remains common in primary care [40], and our imaging finding is consistent with evidence that decision support and targeted reminders can reduce it [41]. A more recent meta-analysis found no overall effect, based on low-certainty evidence, but concluded that educational interventions are unlikely to succeed while organizational and policy-level interventions are more promising [42], a distinction that fits with a tool embedded in the consultation workflow rather than delivered as training. Prescribing, by contrast, has proved responsive to audit and feedback rather than to point-of-care prompts, as in the UK CROP study [43], suggesting that different levers may be required for prescribing than for pathway decisions.

Previous MSK CDSS trials, including SupportPrim [14] and STarT MSK [15], did not assess care quality directly and showed limited influence on clinician decision-making. Our blinded review of records against prespecified indicators offers a feasible method for assessing digital interventions intended to improve care quality and adds to evidence that deimplementation of low-value practice is achievable when workflow and infrastructure are changed, rather than when information alone is supplied [44]. Our findings suggest that a clinician-facing CDSS may improve some aspects of MSK care quality when it is used, although the magnitude of that effect remains uncertain.

### Policy Context

These findings also align with ongoing safety concerns in primary care, particularly those addressed by National Health Service (NHS) England Jess’s Rule, which advises clinicians to “reflect, review, and rethink” when patients reattend three times without diagnostic clarity or documented progression [45]. Repeated prereferral consultations are an established marker of missed diagnostic opportunity in other clinical areas, notably cancer diagnosis [46], although the evidence base for reattendance as a quality marker in MSK care specifically is less developed. In our sample, reattendance without documented change in management was among the most frequent forms of suboptimal care in the control arm and was not observed at CDSS-guided consultations, suggesting that embedded structured support may help operationalize prompts of this kind in routine practice.

### Strengths and Limitations

Strengths of this trial include its real-world design, mixed methods approach, large-scale anonymized EHR data, and use of independent, blinded outcome review. It also incorporated safety, environmental, and economic analyses, enabling a broader assessment of impact. Limitations span multiple domains. In relation to data collection, the follow-up period was short (3 mo); there was an absence of data collection on control clinicians’ information-seeking behavior; and the failure of the patient-reported outcome measure data collection component prevented evaluation of patient-reported outcomes.

The primary outcome should be interpreted cautiously. It rests on a small number of events, and effect estimates derived from sparse data are prone to inflation and imprecision [47], which is reflected in the wide CI around our OR. The composite was also driven by 2 of the 12 indicators; composite outcomes are frequently dominated by a subset of their components, and effects should not be assumed to apply uniformly across the whole composite [48,49]. One of these 2 indicators, repeated attendance without adequate management, involves a degree of subjective clinical judgement, and the appropriateness of imaging for nonspecific low back pain can be difficult to establish from retrospective record review, even with the full consultation notes available. Reviewers were blinded and applied pre-specified indicators, but these constraints limit the precision with which the size of the effect can be estimated.

The attribution rule used in the PP analysis is a further limitation. Suboptimal management in the intervention arm was counted only where it occurred at a consultation in which the CDSS was used, whereas all control consultations contributed. Intervention records therefore had fewer assessable consultations on average (1.13, compared with 1.68 in the control arm). The sensitivity analysis assessing all consultations in both arms showed an effect in the same direction, but smaller and not robust. More broadly, ITT analysis with low uptake estimates the effect of being offered the intervention rather than of receiving it and dilutes the effect of the tool itself [50]; our ITT analysis may additionally have been underpowered, with higher-than-expected ICCs contributing to wide CIs. In relation to the CDSS and its integration, pathway coverage was limited at the time of the study, and the CDSS sat outside the EHR system, so users may have reflected an early-adopter population rather than all primary care clinicians.

Future research should evaluate implementation strategies to increase uptake, particularly those enhancing workflow integration and aligning with incentive structures (such as the advice and guidance scheme). Longer-term evaluations are also needed to assess sustainability, patient outcomes, and wider NHS system impacts. Notably, this trial directly led to the full EMIS and SystmOne integration of the CDSS in January 2025, demonstrating how early digital evaluations can identify barriers that are subsequently resolved through iterative design improvements.

### Conclusions

When used, the CrossCover CDSS was associated with lower rates of nonevidence-based MSK care in UK general practice, although the size of this effect remains uncertain. Benefits were concentrated in referral and imaging decisions, the areas the tool addressed, with little evidence of effect on prescribing or injections. The tool was safe, adaptable, and associated with reductions in both carbon emissions and health care costs, but overall impact was limited by low clinician uptake. Broader adoption will require improved system integration, better alignment with clinical workflows, and the use of incentive frameworks. Further research is needed to evaluate long-term clinical, environmental, economic, and patient-reported outcomes.

## Supplementary material

10.2196/89734 Multimedia Appendix 1 Individual suboptimal management options identified by blinded clinical review of 3-month electronic health records in a random subsample of 120 patients.

10.2196/89734 Checklist 1 CONSORT-EHEALTH checklist.

## Abbreviations

CDSS: clinical decision support system
CONSORT-EHEALTH: Consolidated Standards of Reporting Trials of Electronic and Mobile Health Applications and Online Telehealth.
EHR: electronic health record
FCP: first contact practitioner
GP: general practitioner
ICC: intracluster correlation
ITT: intention-to-treat
MSK: Musculoskeletal
NICE: National Institute for Health and Care Excellence
OR: odds ratio
SRQR: Standards for Reporting Qualitative Research
UKCA: UK Conformity Assessed
