# Supplementary material for: Improving Musculoskeletal Care Quality With a Web-Based Clinical Decision Support System in UK Primary Care: Mixed Methods Cluster-Randomized Controlled Trial
Source: J Med Internet Res. 2026 Oct 15;28:e89734. doi: 10.2196/89734 (PMC13671679; doi:10.2196/89734)
Supplement: Supplementary file 2 [file jmir-v28-e89734-s001.docx]

**Multimedia Appendix 1. Individual sub-optimal management options identified by blinded clinical review of 3-month electronic health records, in a random subsample of 120 patients.**

|  | **Indicator** | **Primary analysis^a^** | | **Sensitivity analysis^b^** | |
| --- | --- | --- | --- | --- | --- |
|  |  | **Control** | **Interv.** | **Control** | **Interv.** |
| 1 | Prescribed NSAIDs for >1 month without concurrent gastroprotection | 1 | 0 | 1 | 0 |
| 2 | Prescribed supplements such as chondroitin or glucosamine | 0 | 0 | 0 | 0 |
| 3 | Prescribed strong opioids for >1 month without indication such as awaiting surgery or fracture | 0 | 1 | 0 | 2 |
| 4 | Prescribed strong gabapentinoids or neuromodulators for >1 month without clinical indication | 1 | 0 | 1 | 1 |
| 5 | Repeat steroid injections into the same joint at intervals shorter than 3 months | 2 | 1 | 2 | 3 |
| 6 | Patients attending multiple (≥3) appointments without adequate management in addition to medications | 5 | 0 | 5 | 0 |
| 7 | Patients issued repeat fit-notes (≥3) without adequate support beyond medication | 2 | 0 | 2 | 2 |
| 8 | Urgent referral to orthopaedic services without clear clinical indication such as red flags | 0 | 0 | 0 | 1 |
| 9 | Simultaneous referral to multiple secondary care specialist services for the same problem | 0 | 0 | 0 | 0 |
| 10 | Repeat imaging requests for the same pain site within 3 months without clear clinical indication | 0 | 0 | 0 | 0 |
| 11 | Imaging requests for non-specific low back pain without clear clinical indication such as red flags | 4 | 0 | 4 | 0 |
| 12 | Imaging requests in patients <45 years old without clear clinical indication such as red flags | 0 | 0 | 0 | 0 |
|  | **Total sub-optimal management options identified, n** | **15** | **2** | **15** | **9^c^** |
|  | **Patients with ≥1 sub-optimal management option, n (%)** | **15 (25.0)** | **2 (3.3)** | **15 (25.0)** | **8 (13.3)** |

NSAIDs: non-steroidal anti-inflammatory drugs.

^a^ Sub-optimal management attributed only where it occurred at a consultation in which the clinical decision support system was used (68 of 105 intervention consultations). All 101 control consultations were assessed.

^b^ All consultations assessed in both arms (101 control, 105 intervention).

^c^ Options exceed patients because one intervention patient triggered two different indicators. To be confirmed.
